# Ab Initio Study of Elastic and Mechanical Properties in FeCrMn Alloys

**DOI:** 10.3390/ma12071129

**Published:** 2019-04-06

**Authors:** Vsevolod I. Razumovskiy, Carola Hahn, Marina Lukas, Lorenz Romaner

**Affiliations:** Materials Center Leoben Forschung GmbH (MCL), Roseggerstraße 12, 8700 Leoben, Austria; Carola.Hahn@mcl.at (C.H.); Marina.Lukas@mcl.at (M.L.); Lorenz.Romaner@mcl.at (L.R.)

**Keywords:** first principles calculations, austenitic steels, mechanical properties, elastic constants, disordered alloys, paramagnetism, solid solution strengthening, stacking fault energy

## Abstract

Mechanical properties of FeCrMn-based steels are of major importance for practical applications. In this work, we investigate mechanical properties of disordered paramagnetic fcc FeCr${}_{10–16}$Mn${}_{12–32}$ alloys using density functional theory. The effects of composition and temperature changes on the magnetic state, elastic properties and stacking fault energies of the alloys are studied. Calculated dependencies of the lattice and elastic constants are used to evaluate the effect of the solid solution strengthening by Mn and Cr using a modified Labusch-Nabarro model and a model for concentrated alloys. The effect of Cr and Mn alloying on the stacking fault energies is calculated and discussed in connection to possible deformation mechanisms.

## 1. Introduction

High strength in steels is typically achieved at the cost of reduced ductility [1]. However, this is not the case for transformation induced plasticity (TRIP) and twinning induced plasticity (TWIP) steels [2,3]. These materials achieve high strength levels through enhanced strain hardening which arises mainly from the interaction of dislocations with stacking faults, twins or ϵ-martensite [3,4]. In addition, the interaction of dislocations with solute atoms gives rise to solid solution strengthening (SSS) which is normally difficult to investigate experimentally as its influence on the strength is difficult to separate from the TWIP and TRIP effects. Therefore, the role of SSS in these classes of steel remains a largely under-investigated phenomenon.

Manganese and chromium are considered to be the main alloying elements in TWIP and TRIP steels [5] characterized by high strength and high ductility. Manganese is known as an austenite stabilizer [6] and is used instead of nickel to lower the costs. Chromium provides corrosion resistance if used above 12 wt.% [7] and increases the nitrogen solubility [6,8], which in its turn is used together with carbon to stabilize the austenitic matrix and also the strength of steels [5,8,9,10]. Presence of C and N in steel makes experimental investigation of the effect of the key alloying elements Mn and Cr on the mechanical properties and possible deformation mechanisms in high strength and high ductility steels a challenging task. The problem arises from the fact that even high purity steels often contain impurities and other allying elements that may have a sizeable effect on SSS and the stacking fault energy (SFE) [11] and thus influence the deformation mechanism [4]. Another problem is the phase stability that is affected by alloying and even small fractions of C and N may change microstructure and phase composition of an austenitic steel [12]. These problems can be avoided in predictive first principles calculations where the crystal structure and chemical composition of an alloy of interest is fully controlled and such properties as SSS and SFE can be investigated rather precisely [13,14,15].

In this paper, we use density functional theory (DFT) to estimate the SSS of FeCrMn alloys based on mechanical models. To this end, we calculate the equilibrium lattice constant and the bulk modulus as a function of Cr and Mn content which serve as input parameters to the existing SSS-models [15,16,17,18,19]. The FeCrMn alloys are magnetically and chemically disordered which poses special requirements to the used DFT methodology. To the best of our knowledge, such ab initio informed modeling of SSS has not been carried out so far. Previous works have relied either on experimental input, see e.g., Ref. [20,21] or on semi-empirical potential modeling, see e.g., Ref. [15]. In addition, we investigate the SFE of FeCrMn alloys, which is a crucial design parameter in austenitic steels [11,22] with regard to the occurring deformation mechanisms (TRIP, TWIP or dislocation slip).

## 2. Methodology

### 2.1. Electronic Structure Calculations

The exact muffin-tin orbital (EMTO) method [23,24] implemented in the Green’s function formalism and combined with the full charge density (FCD) technique [25] has been used in the coherent potential approximation (CPA) [26] calculations of disordered alloys. The paramagnetic state of these alloys has been modeled by the disordered local moment (DLM) model [27,28]. All self-consistent DLM-CPA calculations have been performed using the orbital momentum cut-off of $l_{\max}=3$ for partial waves. The integration over the Brillouin zone has been performed using 37×37×37 Monkhorst-Pack of *k*-points grids [29] for the fcc, 37×37×23 for the hcp and 27×27×13 for the double hexagonal close packed (dhcp) structures, respectively. The core states have been recalculated at each self-consistent iteration. The screening constants for the screened Coulomb interactions have been obtained by the EMTO-LSGF (locally self-consistent Green function) method [30].

### 2.2. Elastic Constants Calculations

Elastic constants and elastic moduli in this work have been calculated following the methodology described in Refs. [31,32,33] in detail. Three independent elastic constants for a cubic system can be represented by the bulk modulus *B*, $C^{\prime }$ = $(C_{11}-C_{12})/2$ and $C_{44}$ [34]. *B* has been calculated using the Birch-Murnaghan fit [35] of the equation of state (EQOS). The EQOS has been calculated using the Wigner-Seiz radii (RWS) of the fcc alloys from 2.50 to 2.70 a.u. with a step of 0.02 a.u. $C^{\prime }$ and $C_{44}$ have been calculated using volume conserving orthorhombic and monoclinic strains, respectively [31]. The value of distortion *x* in $C^{\prime }$ and $C_{44}$ calculations has been varied from zero (for the equilibrium state) to 0.05 with a step size of 0.01, in accordance with Mehl et al.’s prescription [31].

### 2.3. Solute Solution Strengthening Model

We use two different approaches for the calculation of SSS, the model by Labusch-Nabarro (LN) [16,20,36,37,38], which we call the Labusch-Nabarro model in the following, and the model by Varvenne et al. [15], named as the VC model in the following.

Within the LN model, solid solution strengthening for a multi-component alloy can be calculated as [20,37,38]

(1) $$\tau_{LN}=AG\sum_{n}{\left({\epsilon_{L}^{n}}^{2}c_{n}\right)}^{2/3},$$

where $c_{n}$ is the concentration of solute n and *G* is the isotropic shear modulus which we obtain from the elastic constants by Voigt averaging. The constant factor *A* is equal to $\left(10^{1/3}/2\right)\cdot 120^{-4/3}$ assuming that the parameter *w* in the LN model is equal to 5b [39] where *b* is the Burgers vector.

Misfit parameters treat the two main types of interactions between dislocations and solute atoms. The first misfit parameter ($\epsilon_{b}^{n}$, Equation (2)) arises due to different sizes of the alloying elements compared to the matrix elements, which leads to the strain field around solute atoms (lattice misfit). The strain field of a dislocation interacts with the strain field of solute atoms, more energy is required to move the dislocation further (paraelastic interaction) [40,41].

(2) $$\epsilon_{b}^{n}=\frac{1}{b}\frac{db}{dc_{n}}$$

The second misfit parameter ($\epsilon_{G}^{n}$, Equation (3)) arises because solute atoms have a different shear modulus than the matrix atoms (modulus misfit). Therefore, dislocations containing solute atoms have a different elastic energy compared to dislocations containing only matrix atoms and more energy is required for the movement of a dislocation containing solute atoms (dielastic interaction) [40,41].

(3) $$\epsilon_{G}^{n}=\frac{1}{G}\frac{dG}{dc_{n}}$$

The two misfit parameters are combined into the single misfit parameter $\epsilon_{L}^{n}$ [17,21]:

(4) $$\epsilon_{L}^{n}=\sqrt{{\left(\epsilon_{G^{\prime }}^{n}\right)}^{2}+{\left(\alpha \epsilon_{b}^{n}\right)}^{2}},$$

where α is a constant, with α≥ 16 in the case of edge dislocations and α≤ 16 in the case of screw dislocations [17] and $\epsilon_{G^{\prime }}^{n}=\epsilon_{G}^{n}/\left(1+0.5|\epsilon_{G}^{n}|\right)$. In this work, we adopt α=16.

In the VC model [15], SSS is described as

(5) $$\tau_{VC}=0.051\alpha^{\prime -1/3}G{\left(\frac{1+\nu }{1-\nu }\right)}^{4/3}f_{1}{\left(\frac{\sum_{n}c_{n}\Delta V_{n}^{2}}{b^{6}}\right)}^{2/3}$$

where $\alpha^{\prime }=0.123$, ν is the Poisson ratio, $f_{1}$ is a parameter which equals to 0.35 provided the SFE is below 100 mJ/m${}^{2}$, and $\Delta V_{n}$ is the volume mismatch between the effective medium and element *n*. The volume mismatch is the key quantity for strengthening. In the VC model, no distinction between lattice misfit and modulus misfit is made.

The quantities $\tau_{LN}$, $\tau_{VC}$ correspond to the critical resolved shear stress (CRSS) required to move dislocations through the solid solution at 0 K. For the VC model we also calculate the temperature dependence of $\tau_{VC}$ following the method outlined in Ref. [15].

### 2.4. Stacking Fault Energy Calculations

The intrinsic stacking-fault (SF) is one of the simplest planar defects of the crystal lattice. It is characterized by a fault in the usual ABC planar stacking sequence of the fcc structure, ...ABCAB|ABC..., which resembles locally the stacking sequence of the hcp structure. In the framework of the axial Ising model (AIM) [42,43], the SFE γ can be determined in terms of the total energies of the fcc, hcp, and dhcp structures:

(6) $$\gamma \left(T\right)=F_{hcp}\left(T\right)+2F_{dhcp}\left(T\right)-3F_{fcc}\left(T\right),$$

where $F_{fcc}$, $F_{hcp}$ and $F_{dhcp}$ are the free energies of the fcc, hcp and dhcp phases and *T* is temperature. This formulation accounts for the interactions between the next nearest neighbor stacking plains and is knowns as the axial next nearest neighbor Ising model (ANNNI). The ANNNI has been shown to be a reasonable choice in terms of the accuracy and computational costs of required DFT calculation in the case of fcc Fe and FeMn alloys [13] and therefore has been selected as the method of choice in our study.

### 2.5. Finite Temperature Calculations

The contributionsfrom electronic excitations [44] and the magnetic entropy [45] have been taken into account in direct self-consistent DFT calculations. The magnetic entropy contribution due to longitudinal spin fluctuations [45] has been accounted in the following form [46]

(7) $$S_{lsf}=log\left(\langle m_{i}\rangle \right),$$

where $\langle m_{i}\rangle$ is the average magnitude of the magnetic moment of the i-th alloy component, which is the result of the corresponding DFT self-consistent calculations.

The vibrational contribution to the free energy in the SFE calculations (Equation (6)) has been considered implicitly by employing the thermal lattice expansion taken from the Debye-Grüneisen model [47,48]. As input parameters for the model, we have used the 0 K equilibrium DFT data presented in Section 3.

## 3. Results and Discussion

**Figure 1:**
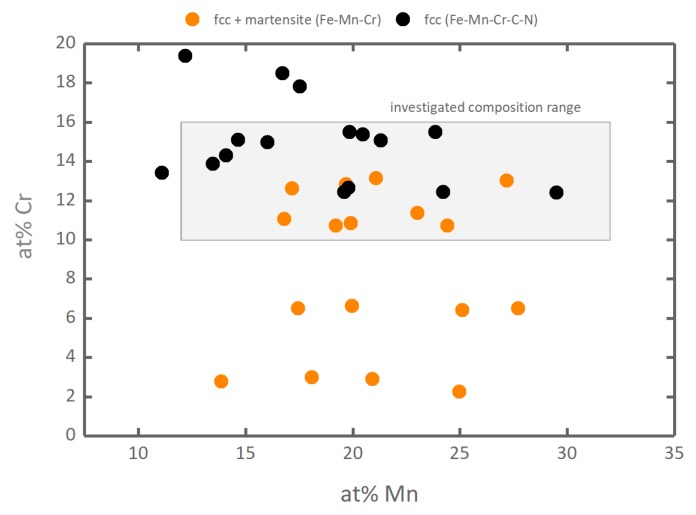
Experimental alloy compositions and their respective phases. Fe-Mn-Cr alloys (orange) are only experimentally obtained with a mixture of fcc and martensite [12], whereas Fe-Mn-Cr-C-N alloys (black) can be experimentally obtained with a fully austenitic microstructure [8,49,50,51,52,53]. For a detailed listing of the references, the reader is referred to the supplemental material.

Paramagnetic (PM) FeMnCr alloys within the compositional range for Mn varying from 12 to 32 at.% and Cr varying from 10 to 16 at.% have been selected for the investigation. This compositional range is of special interest, because it is not possible to experimentally study the influence of Mn and Cr on the SSS and SFE in an austenitic steel within this range, since it is experimentally not possible to obtain a fully austenitic microstructure with just Fe, Mn and Cr in the defined ranges. To experimentally obtain a fully austenitic microstructure for comparable amounts of Mn and Cr, additional alloying with C and N is required (see Figure 1). However, alloying with the interstitial elements C and N can superpose the effect of Mn and Cr. In what follows, all compositions will be given in atomic percent and all results will refer to these compositional ranges unless specified otherwise.

### 3.1. Equation of State

**Figure 2:**
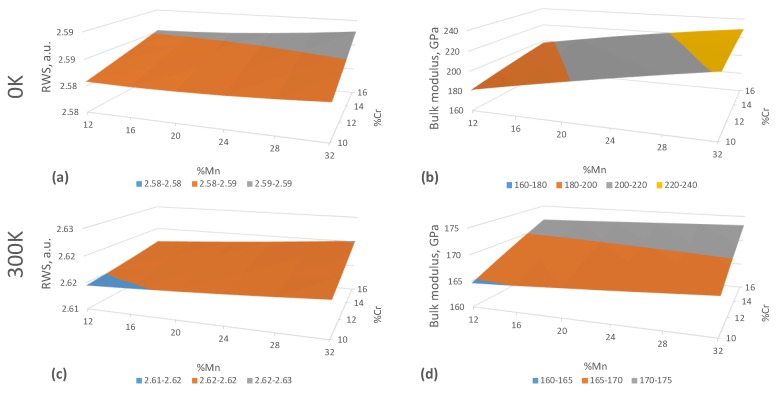
Equilibrium Wigner-Seitz radii (RWS, in a.u.) at 0 K (**a**) and at 300 K (**c**) and the bulk modulus at 0 K (**b**) and at 300 K (**d**) in PM fcc random FeMnCr alloys.

As the first step in our investigation, we have calculated the equilibrium Wigner-Seiz radius and the bulk modulus *B* in FeCr${}_{10–16}$Mn${}_{12–32}$ alloys shown in Figure 2a,c. The results on the bulk modulus at 0 K provide values ranging from 181 GPa for FeCr${}_{10}$Mn${}_{12}$ to 229 GPa for FeCr${}_{16}$Mn${}_{32}$ alloy. These values overestimate typical experimental *B* room temperature values for the austenitic Cr- and Mn-containing steels that vary in the range of 158–167 GPa [54,55].

However, as our further calculations show, the DFT results substantially change if the effect of temperature is included in the calculations even at room temperature. The DFT calculations at 300 K including electron and magnetic entropy thermal excitations yield about 0.04 a.u. higher RWS and lower *B* in the range from 165 GPa for FeCr${}_{10}$Mn${}_{12}$ to 173 GPa for FeCr${}_{16}$Mn${}_{32}$ alloy. The *B* concentration dependence remains the same at both 0 K and 300 K but the results at 300 K provide much better agreement with the availible experimental data (158–167 GPa [54,55]). The observed trend that *B* increases with increasing Mn content, also agrees well with the theoretical results of Ref. [56].

**Figure 3:**
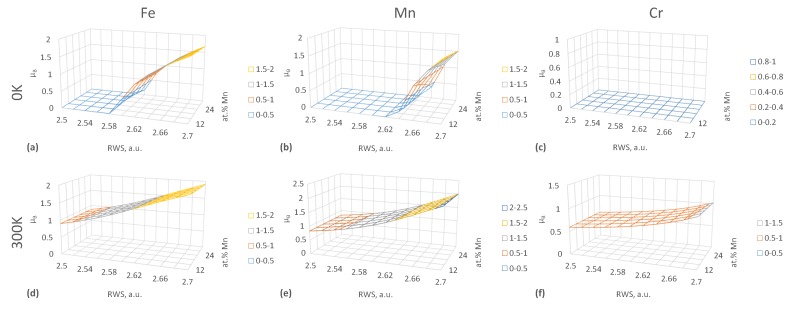
Magnetic moments (in $\mu_{B}$) in FeCr${}_{10}$Mn${}_{12–32}$ PM alloys as a function of Wigner-Seiz radii and Mn concentration. (**a**) Magnetic moments on Fe atoms at 0 K. (**b**) Magnetic moments on Mn atoms at 0 K. (**c**) Magnetic moments on Cr atoms at 0 K. (**d**) Magnetic moments on Fe atoms at 300 K. (**e**) Magnetic moments on Mn atoms at 300 K. (**f**) Magnetic moments on Cr atoms at 300 K.

As a more detailed inspection of our DFT results shows, the main reason for the sizeable increase of RWS and decrease of *B* is the enhancement of the magnetic moment (MMOM) due to longitudinal spin fluctuations (LSF) in the 300 K calculations. The evolution of local MMOMs on atoms in FeCr${}_{10}$Mn${}_{12–32}$ paramagnetic alloys as a function of RWS, Mn composition and temperature are shown in Figure 3 as an example. The results show that there is a so-called magnetic high-spin HS to low-spin LS transition near the equilibrium-RWS at both Fe and Mn atoms, which may complicate any analytical operations with the EQOS [57]. Here, we refer to the LS state as the DLM magnetic state with zero local magnetic moment and to the HS state as the DLM state with a non-zero local magnetic moment. LSF at 300 K give rise to magnetic moments on all atoms at low RWS and on Cr atoms in general, where MMOMs are zero at 0 K. LSF enhance also the MMOM at higher RWS for all Fe, Mn and Cr atoms. The high-spin to low-spin transition also disappears which, along with increased equilibrium-RWS values at 300 K (Figure 2), leads to the decrease of *B* with increasing temperature. Our results predict qualitatively the same picture for all FeCr${}_{10–16}$Mn${}_{12–32}$ alloy compositions.

### 3.2. Elastic Constants

The $C^{\prime }$ and $C_{44}$ elastic constants in a cubic system form a full set of independent elastic constants along with the bulk modulus *B* calculated in the previous section (Section 2.2). As the next step of our investigation, we have calculated $C^{\prime }$ and $C_{44}$ in PM FeCr${}_{10–16}$Mn${}_{12–32}$ alloys at 300 K using the equilibrium-RWS results from Section 3.1.

**Figure 4:**
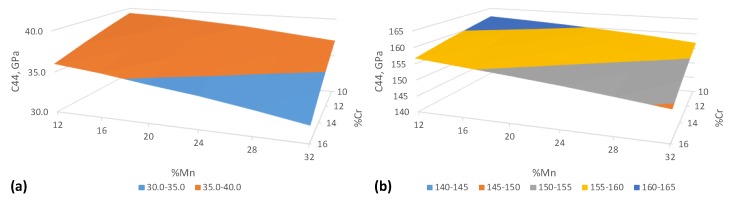
Elastic constants of FeCr${}_{10–16}$Mn${}_{12–32}$ alloys: (**a**) $C^{\prime }$; (**b**) $C_{44}$ at 300 K in GPa.

The calculated elastic constants are shown in Figure 4 as a function of Cr and Mn content. The dependence on the compositional changes in the selected range is also linear as in the case of *B*. $C^{\prime }$ increases from 32 (FeCr${}_{16}$Mn${}_{32}$ ) to 39.3 GPa (FeCr${}_{10}$Mn${}_{12}$) as the concentration of Mn and Cr decreases in the alloy. We see the same behaviour in the case of $C_{44}$ which increases from 149 to 162 GPa in the same compositional range. These values agree well with the experimental and theoretical results on Fe-Mn and Fe-Cr-Mn-Ni alloy systems yielding $C_{44}$ in a range from 122 to 140 GPa and $C^{\prime }$ from 25 to 38 GPa for various alloy compositions [54,55,56,58]. The elastic constants do not exhibit any peculiarities and have a virtually linear concentration dependence in the whole explored compositional range which is very advantageous for analytical modeling of the solid solution strengthening described in Section 3.4.

### 3.3. Stacking Fault Energies

Determination of the exact SFE values and the effects of the alloying elements on the SFE is a challenging task. Numerous experimental studies of the effect of alloying elements on SFEs in steels, recently reviewed in Refs. [59,60], provide qualitatively and quantitatively different results. For instance, in Ref. [61] it was concluded that Cr and Mn reduce the SFE in some austenitic steels. A neural-network analysis over a large literature database presented in Ref. [60] associates Mn with a strong increase of the SFE, whereas Cr has no influence at all. These contradicting results show that all alloying elements can influence the SFE and further research is required to understand the influence of individual alloying elements on the SFE.

**Figure 5:**
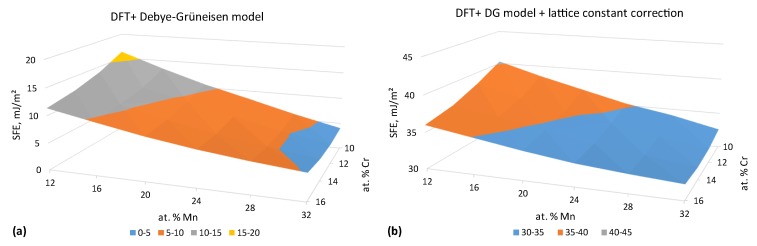
Intrinsic stacking fault energy of the PM FeCrMn alloys at 300 K. (**a**) DFT and Debye-Grüneisen model calculations. (**b**) DFT and Debye-Grüneisen model calculations corrected to the absolute lattice constant value difference between theoretical results and the experiment [66].

**Figure 6:**
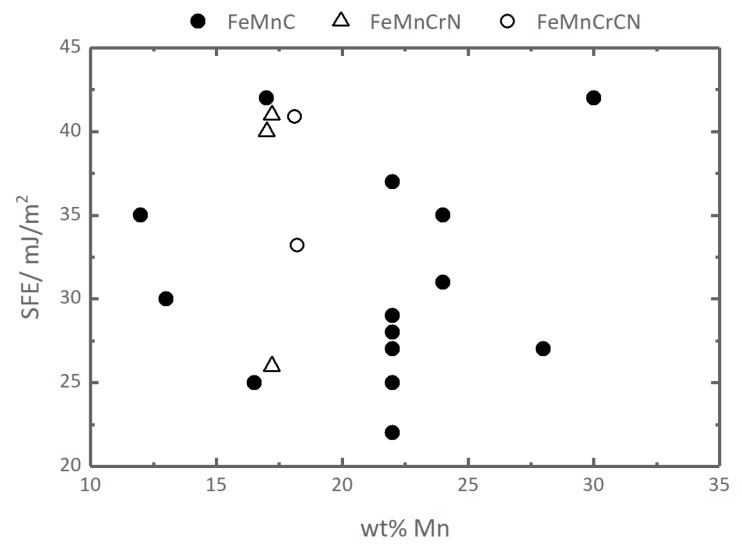
SFE of various austenitic steels. FeMnC: [67,68,69,70,71,72]; FeMnCrN: [71,73]; FeMnCrCN: [74]. For exact chemical compositions see Table A1 in Appendix A.

Following the methodology described in Section 2.4, we have calculated the SFEs in PM FeCr${}_{10–16}$Mn${}_{12–32}$ alloys. The results, based on DFT calculations with the magnetic entropy contribution from the LSF and the effect of lattice expansion taken into account via the Debye-Grüneisen model [47], are shown in Figure 5a. The results of DFT calculations with theoretical lattice constants are known to underestimate the SFE [13,14,57,62], due to the underestimation of the equilibrium lattice constant of Fe and its alloys in DFT in general [13,32,63,64,65]. This problem can be solved by using the experimental lattice parameters in DFT+LSF calculations of SFEs in paramagnetic Fe-base fcc alloys [13,57]. We have not found sufficient experimental data on the lattice parameters of the alloys of our interest and used the Debye-Grüneisen (DG) model data on lattice expansion increased by a constant value of 0.027 Å corresponding to the difference between the DFT+DG and experimental data for the lattice constants of FeCr${}_{18}$Mn${}_{10}$ steel [66]. The SFE values calculated with the corrected lattice parameters are presented in Figure 5b. The comparison to theSFEs calculated without the correction (Figure 5a) reveals an about 25 mJ/m${}^{2}$ increase of the SFEs with respect to uncorrected data, which is in qualitative agreement with previously reported works on FeMn steels [13,57]. The SFE values presented in Figure 5b suggest that both Mn and Cr alloying elements reduce the SFE in FeCrMn alloys. The absolute values of the SFE in our calculations vary from about 30 to 40 mJ/m${}^{2}$ is a typical range for the SFEs in austenitic steels (Figure 6).

The absolute values of the SFEs are often used to estimate possible deformation modes in austenitic steels [4,59,75]. A good summary of the SFE-ranges and the related deformation modes is given in Ref. [4]. According to the present results, the SFEs in FeCr${}_{10–16}$Mn${}_{12–32}$ alloys fall in the range between 30 to 40 mJ/m${}^{2}$, which is related with twinning in literature [4,59].

### 3.4. Solid Solution Strengthening

The compositional dependencies of the lattice constants and shear modulus obtained in the previous sections can be directly used for modeling of SSS in FeCrMn alloys using the LN model or the VC model as described in Section 2.3. For the latter the volumes $V_{n}$ (where n= Fe, Cr or Mn) were extracted by fitting the volume of the alloy, *V*, with $V=\Sigma c_{n}V_{n}$, and $V_{n}$ treated as fit parameters. The results are $V_{Fe}=11.00$ Å${}^{3}$, $V_{Mn}=11.17$ Å${}^{3}$, $V_{Cr}=11.67$ Å${}^{3}$. Fe, hence, has the smallest volume, followed by Mn and then Cr, where the difference is strongest for Cr. The ordering is according to the periodic table and reveals that larger band filling leads to smaller volumes. Please note that the relative ordering of volumes was different in Ref. [15], where volumes where extracted from experimental data on austenitic high-entropy alloys. In that work, Mn exhibited a much higher volume compared to Cr. This could either be related to the Ni content in those materials, or by the specific fitting procedure used to extract the volumes in Ref. [15]. We leave a more detailed investigations of this subject to future work.

The concentration dependencies of the lattice parameter and elastic constants were also used to calculate the lattice ($\epsilon_{b}$) and the shear modulus ($\epsilon_{G}$) mismatch contributions needed for the LN model as defined by Equations (2) and (3). Due to virtually linear concentration dependence of the lattice parameter and shear modulus on the concentration (see Figure 2 and Figure 4), $\epsilon_{b}$ and $\epsilon_{G}$ remain essentially constant within the whole range of considered alloy compositions (Cr 10 to 16 at.% and Mn 12 to 32 at.%). The obtained results for $\epsilon_{b}$ and $\epsilon_{G}$ are presented in Table 1.

The DFT data on $\epsilon_{b}$ presented in the table can be compared with the analogous data from Ref. [21]. For Cr, the values are in reasonable agreement while for Mn there exists a rather pronounced disagreement. We attribute this to the rather crude approximation used in Ref. [21] for evaluation of $\epsilon_{b}$, where $\epsilon_{b}$ was estimated as a difference between the atomic volume of bcc (instead of fcc) iron and the atomic volume of a solvent in its reference state. In fact, the experimental investigation of the lattice constants in concentrated Mn-rich austenitic steels [76] has shown that the effect of Mn on the lattice parameter is non-linear being rather strong at low Mn contents (up to 6 at.% of Mn) but leveling out to virtually no effect for the compositions with more than 6% Mn. This result agrees well with the results of our DFT calculations.

The modulus mismatch $\epsilon_{G}^{\prime }$ of both Cr and Mn atoms shown in Table 1 is 1–2 orders of magnitude larger than that of the lattice ($\epsilon_{b}$). Therefore, also $\epsilon_{L}$ is dominated by this contribution in agreement with the conclusion drawn in Ref. [17] for substitutional fcc Cu alloys.

**Figure 7:**
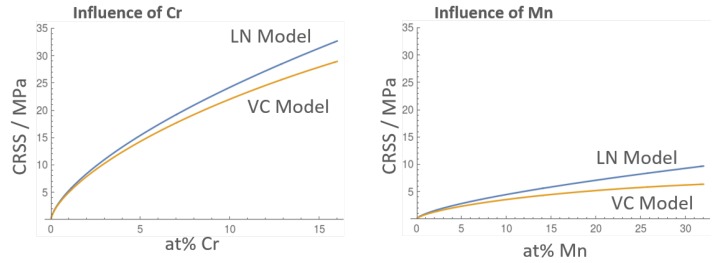
Solid solution strengthening in Fe for separate Cr and Mn alloying.

Based on the misfit quantities $\tau_{LN}$ and $\tau_{VC}$ can be calculated. Here, we would like to remind that the quantities $\tau_{LN}$, $\tau_{VC}$ correspond to the CRSS required to move dislocations through the solid solution. In Figure 7 the results are shown for both models for the binaries. For clarity, the concentration range is extended to $c_{n}=0$, i.e., outside of the concentration regime covered by the DFT calculations. The figure shows that addition of both Cr and Mn alloying elements increases CRSS throughout the whole compositional range. We can see that the models largely agree with each other, with the VC model yielding slightly smaller values. In view of the substantial difference in the derivation of the models, the agreement is rather satisfying and gives confidence about their reliability.

**Figure 8:**
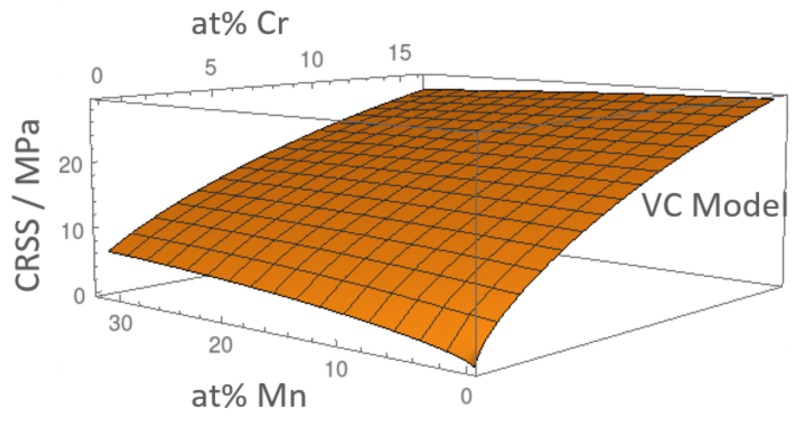
Solid solution strengthening in FeCrMn alloys with the VC model.

The overall SSS effect in FeCrMn alloys is shown in Figure 8. We can see that within the compositional range of interest, the SSS is maximal for $c_{Cr}=16$ at.% where $\tau_{VC}=28$ MPa and minimal for $c_{Mn}=12$ at.% where $\tau_{VC}=21$ MPa. Surprisingly, raising the Mn content reduces $\tau_{VC}$ in the compositional range of interest of the ternary in contrast to the behavior of the binary. This can be explained by the fact that the volume of Mn is intermediate to Fe and Cr and, hence, closest to the effective medium. Replacing an Fe- or Cr-atom by Mn, therefore, leads to a more homogeneous alloy with a smaller SSS contribution. As a result, the highest strength can be achieved by maximizing the Cr content and minimizing the Mn content.

**Figure 9:**
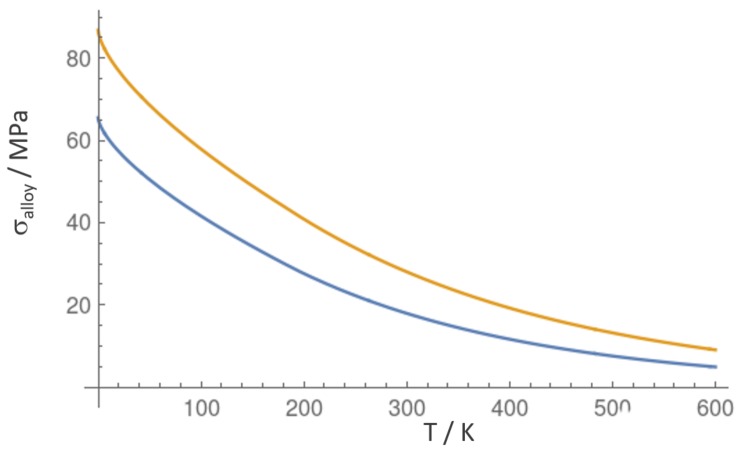
Yield stress versus temperature for $c_{Cr}=16$ at.% (orange, strongest alloy) and $c_{Mn}=12$ at.% (blue, softest alloy).

The temperature dependence of the SSS of polycrystalline austenitic FeMnCr, $\sigma_{alloy}$, is shown in Figure 9 for the strongest and softest alloy composition. At 0 K, $\sigma_{alloy}$ corresponds to $\tau_{VC}$ multiplied by the Taylor factor of 3.06, therefore, strengthening amounts between 87 and 65 MPa. At room temperature, the strengthening contribution is reduced to about 1/3 of this value, i.e., 18 and 29 GPa.

Thus, SSS is relatively moderate for the concentration range of interest. In other ternary alloys, the equivalent SSS was found to be sizably higher, around 75–100 MPa [15]. This difference is explained by the relatively low lattice mismatch between Fe and Mn in our case. Cr, which would be a more effective strengthener, is added at relatively small concentrations because Cr is a ferrite-stabilizing element and thus more likely than Mn (austenite-stabilizer) to induce a phase transformation [77].

## 4. Conclusions

First principles calculations of the equilibrium elastic properties of paramagnetic FeCr${}_{10–16}$Mn${}_{12–32}$ alloys suggest that the lattice constants and the elastic constants change linearly with composition within the considered compositional range. The lattice constants and the bulk modulus linearly increase with increasing Cr and Mn content while the shear elastic constants $C^{\prime }$ and $C_{44}$ decrease linearly. In terms of SSS, Cr additions are much more effective than Mn additions due to the much higher volume of the former compared to the latter. The absolute value of the SSS at room temperature for polycrystalline austenite, $\sigma_{alloy}$, ranges from 18 (low Cr and high Mn content) to 29 MPa (high Cr and low Mn content).

DFT calculations predict that alloying with both Mn and Cr should reduce the SFE in FeCr${}_{10–16}$Mn${}_{12–32}$ alloys. The absolute values of the SFE in our calculations varying from about 30 to 40 mJ/m${}^{2}$ suggest that these materials are a subject to the TWIP deformation mechanism.

## Figures and Tables

**Table 1:** Atomic size misfit $\epsilon_{b}$ and the modulus mismatch $\epsilon_{G}^{\prime }$ for PM FeCr${}_{10–16}$Mn${}_{12–32}$-base alloys.

Element	$\epsilon_{b}$	$\epsilon_{b}$ [21]	$\epsilon_{G}^{\prime }$	$|\epsilon_{L}|$
Cr	0.020	0.031	−0.53	0.63
Mn	0.005	0.073	−0.15	0.17

## Supplementary Materials

Supplementary materials can be found at https://www.mdpi.com/1996-1944/12/7/1129/s1.

## Abbreviations

The following abbreviations are used in this manuscript:

ANNNI	axial next nearest neighbor Ising model
CPA	coherent potential approximation
CRSS	critical resolved shear stress
DFT	density functional theory
DG	Debye-Grüneisen
dhcp	double hexagonal close packed
DLM	disordered local moment
EMTO	exact muffin-tin orbital
EQOS	equation of state
FCD	full charge density
LN	Labusch-Nabarro model
LSF	longitudinal spin fluctuations
LSGF	locally self-consistent Green function method
MMOM	magnetic moment
PM	paramagnetic
PN	Peierls-Nabarro model
RWS	Wigner-Seiz radius
SFE	stacking fault energy
SSS	solid solution strengthening
TRIP	transformation induced plasticity
TWIP	twinning induced plasticity
VC	Varvenne-Curtin model

## Appendix A. Literature Data for SFE

The exact chemical compositions related to the data points of Figure 6 and Figure 5 are given in Table A1 and Table A2.

**Table A1:** Austenitic steels, their chemical composition and respective SFE, data belong to Figure 6.

Concept	Mn [wt.%]	Cr [wt.%]	C [wt.%]	N [wt.%]	Si [wt.%]	SFE [mJ/m${}^{2}$]	Methode	Ref.
FeMnC	22	0	0.60	0	0	22	Graph	[67]
FeMnC	22	0	0.60	0	0	37	Calculation	[68]
FeMnC	17	0	0.95	0	0	42	Calculation	[68]
FeMnC	30	0	0.50	0	0	42	Calculation	[68]
FeMnC	17	0	0.81	0	0	25	Calculation	[69]
FeMnC	22	0	0.60	0	0	27	Calculation	[70]
FeMnC	28	0	0.30	0	0	27	Calculation	[70]
FeMnC	24	0	0.70	0	0	35	Calculation	[70]
FeMnC	24	0	0.60	0	0	31	Calculation	[70]
FeMnC	12	0	1.20	0	0	35	Calculation	[70]
FeMnC	22	0	0.03	0	0	29	Calculation	[71]
FeMnC	22	0	0.33	0	0	25	Calculation	[71]
FeMnC	22	0	0.69	0	0	28	Calculation	[71]
FeMnC	13	0	1.30	0	0	30	Literature	[72]
FeMnCrN	17	15	0	0.80	0	41	Calculation	[71]
FeMnCrN	17	15	0	0.23	0	26	Measurement	[73]
FeMnCrN	17	15	0	0.80	0	41	Measurement	[73]
FeMnCrCN	18	7	0.59	0.29	0.56	33	Calculation	[74]
FeMnCrCN	18	7	0.78	0.17	0.47	41	Calculation	[74]

**Table A2:** Austenitic steels, their chemical composition and respective SFE obtained in DFT calculations at 300 K, data belong to Figure 5b.

Cr [at.%]	Mn [at.%]	SFE [mJ/m${}^{2}$]
10	12	40.3
10	16	38.5
10	20	36.9
10	24	35.2
10	28	34.1
10	32	32.6
12	12	38.4
12	16	36.7
12	20	35.4
12	24	34.3
12	28	33.0
12	32	31.9
14	12	36.8
14	16	35.8
14	20	34.6
14	24	33.4
14	28	32.6
14	32	31.7
16	12	36.0
16	16	35.0
16	20	33.9
16	24	33.1
16	28	32.5
16	32	32.1

## Appendix B. Fit Parameters

The lattice constants (in Å), the bulk modulus (in GPa), the elastic constants $C_{44}$ (in GPa) and $C^{\prime }$ (in GPa) as a function of atomic fraction of Mn ($c_{Mn}$) and Cr ($c_{Cr}$) have been fitted with an analytical function of a form

(A1) $$z=Ac_{Cr}+Bc_{Mn}+C.$$

The fit parameters for each of the aforementioned quantities are listed in Table A3 below.

**Table A3:** Fit parameters *A*, *B* and *C* for the lattice constants ($a_{lat}$), the bulk modulus (*B*), the elastic constants $C_{44}$ (in GPa) and $C^{\prime }$ (in GPa) as they appear in Equation (A1).

Quantity	*A*	*B*	*C*
$a_{lat}$	1.8019	6.9418	3.5306
*B*	0.0969	1.1234	152.4966
$C_{44}$	−0.3023	−1.0925	177.4827
$C^{\prime }$	−0.1534	−0.7025	48.8365
