# Supplementary material for: Ab Initio Study of Elastic and Mechanical Properties in FeCrMn Alloys
Source: Materials (Basel). 2019 Apr 6;12(7):1129. doi: 10.3390/ma12071129 (PMC6480156; doi:10.3390/ma12071129)
Supplement: Supplementary file 1 [file materials-12-01129-s001.pdf]

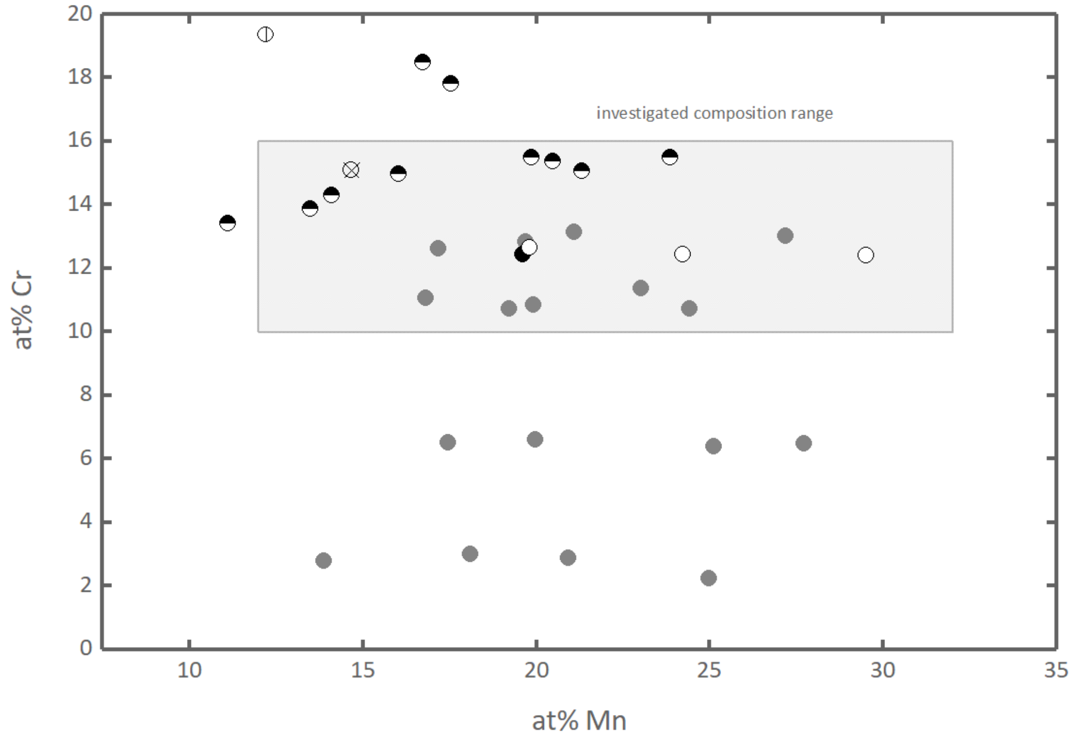

fcc + martensite (Fe-Mn-Cr)

- ● F. Malamud et al., Mater. Des. Vol. 139 (2018) pp.314–323, DOI:10.1016/j.matdes.2017.11.017

fcc (Fe-Mn-Cr-C-N)

- ⦶ M. Xu et al., Adv. Mater. Res. Vols. 146-147 (2010) pp 26-33, DOI:10.4028/www.scientific.net/AMR.146-147.26
- ◒ J.C. Rawers, J. Mater. Sci. Vol. 43 (2008) pp. 3618–3624, DOI:10.1007/s10853-008-2576-3
- ⦻ L. Mosecker et al., Mater. Sci. Eng. A. Vol. 642 (2015) pp. 71–83, DOI:10.1016/j.msea.2015.06.047
- ● L.M. Roncery et al., Metall. Mater. Trans. A Vol. 41 (2010) pp. 2471–2479, DOI:10.1007/s11661-010-0334-z
- ○ L.M. Roncery et al., steel research int. Vol 83 (2012) pp.307-314, DOI:10.1002/srin.201100316
